# Pregnant Women’s Anxiety and Depression Symptoms and Influence Factors in the COVID-19 Pandemic in Changzhou, China

**DOI:** 10.3389/fpsyg.2022.855545

**Published:** 2022-05-26

**Authors:** Li Wang, Ni Yang, Hua Zhou, Xueqin Mao, Ying Zhou

**Affiliations:** ^1^Changzhou Maternal and Child Health Care Hospital, Changzhou, China; ^2^Department of Obstetrics and Gynecology, Nanjing Medical University, Nanjing, China; ^3^Changzhou Center for Disease Control and Prevention, Changzhou, China

**Keywords:** pregnant women, anxiety, depression, influence factor, COVID-19

## Abstract

**Background:**

With the coronavirus disease 2019 (COVID-19) pandemic, pregnant women’s psychological conditions have become a global challenge. The aim of the study was to identify the prevalence of anxiety and depression symptoms and analyze their influence factors among pregnant women in Changzhou, China during the COVID-19 pandemic and provide them with appropriate healthcare strategies.

**Methods:**

Participants were 681 pregnant women who visited various obstetrics and gynecology clinics in Changzhou, China between 25 February 2020 and 7 March 2020. They were asked to complete an online survey. The Generalized Anxiety Disorder Scale-7 (GAD-7) and Patient Health Questionnaire-9 (PHQ-9) were used to detect anxious and depressive symptoms. The chi-squared test and multivariate logistic regression analysis were carried out to examine the associated factors in these groups.

**Results:**

Overall, the prevalence rates of anxiety and depression symptoms among pregnant women were 31.72 and 36.12%, respectively, but most of them were mild. Having an irregular diet with poor subjective sleep quality, perceiving little family support, spending too much time on news related to the coronavirus, and having severe subjective life impact by the coronavirus were related to anxiety and depression symptoms. In addition, lack of physical exercise and exposure to electronic screens over 5 h per day were associated with depression symptoms.

**Conclusion:**

Pregnant women with an irregular diet, poor sleep quality, little family support, excessive attention to COVID-19 news, and lives impacted by the coronavirus severely are at high risk for anxiety and depression symptoms during the COVID-19 pandemic. This indicates that targeted measures to address mental health in pregnant women during the pandemic period are needed.

## Introduction

The coronavirus disease 2019 (COVID-19) pandemic, first reported in December 2019 and declared as a COVID-19 pandemic in January 2020, has become a novel global health emergency (Zhu et al., 2020). Although the COVID-19 vaccines effectively prevent infection, some cases of infections have been found post-vaccination (Vaishya et al., 2021). Moreover, a specific drug therapy has not been found or developed, so social isolation is still the principal measure to block the transmission of the disease (Ma et al., 2020). China has adopted the general policy of “dynamic clear.” Public health measures, such as wearing masks, washing hands frequently, and social isolation (isolating infected cases, limiting gathering and travel), are still our prevention methods. Thus far, COVID-19 cases continue to break out all over the world, and there is no indication that the COVID-19 pandemic will end in the short term. This global health emergency has a profound influence on many aspects of society (Ayittey et al., 2020). With the spread of the COVID-19 pandemic, psychological crises have become a global challenge, particularly for highly vulnerable populations (Qiu et al., 2020). Hence, identifying these population groups and guaranteeing them appropriate healthcare treatment are necessary (Hossain et al., 2020).

In immune system suppression, there is an increased risk of severe complications, uncertainties in antenatal care, and increased concern about vertical transmission to the fetus (Lindheimer and Cunningham, 2014; Morhart et al., 2021). Pregnant women may be one of the groups particularly vulnerable to infections. Moreover, previous studies show that the risk of negative psychological symptoms among pregnant women has increased with infection outbreaks (Filgueiras Meireles et al., 2017). Chinese researchers reported that depression in pregnant women with depression increased significantly after the declaration of the COVID-19 pandemic (Wu et al., 2020). Maternal mental health is very important to the psychological and physical health of the mother and the fetus (Wisner et al., 2019). Previous studies noted that severe perceived stress was significantly associated with shorter gestational age, lower birth weight, lower Apgar scores, and a higher risk of complications, such as delayed neurodevelopment (Van den Bergh et al., 2020). In addition, recent studies demonstrated that pregnant women infected with COVID-19 were more likely to experience perinatal death, preterm delivery, and low birthweight (Chen et al., 2020; Du et al., 2021). Thus, pregnant women have special needs for preventive mental health measures.

Given the above considerations, the aim of this study was to examine the impact of the COVID-19 outbreak on the prevalence of anxiety and depression symptoms and the potential influencing factors among pregnant women.

## Materials and Methods

### Study Design and Participants

We conducted a web-based cross-sectional survey in Changzhou city from 25 February to 7 March 2020. Changzhou is located in the economically developed eastern part of China with a large population density. It reported 51 confirmed cases from 25 January to 8 March 2020. A convenience sample was recruited for this study. The participants were local pregnant women who visited obstetrics and gynecology clinics in Changzhou Maternity and Child Healthcare Hospital and Community Health Service Centers of Changzhou for registration of their maternal healthcare system card and antenatal examination. They were asked to complete in writing an online questionnaire with demographic data and emotional evaluation scales. For supervising regular antenatal examinations and distributing maternal healthcare knowledge, pregnant women in Changzhou are invited to join WeChat groups, such as a maternal healthcare system card registration group or a pregnant woman school group. A quick response code (QR code) and relevant link to the questionnaire address were sent on the internet through the abovementioned WeChat groups and the WeChat public account of Changzhou Maternity and Child Healthcare Hospital, whose majority of followers are women of childbearing age. To increase the recruitment of potential participants, every participant could receive a report on their mental health and related advice after submitting the evaluation. In addition, the psychological hotline of the Women’s Psychological Clinic of Changzhou Maternity and Child Healthcare Hospital was available to participants for free. The exclusion criteria for participants were women with mental disorders diagnosed previously and those who spent less than 5 min to fill out the questionnaire. The survey objectives, contents, associated risks and benefits, and options for voluntary participation and informed consent were provided at the beginning of the questionnaire. Participants selected the option and then got access to the questionnaire. The study protocol was approved by the Ethics Committee of Changzhou Maternal and Child Health Care Hospital.

### Measurement

Sociodemographic variables, daily behaviors, family status, coronavirus-related events, and a measure of anxiety and depression symptoms were included in a self-administrated questionnaire. There were logical relationships between items to avoid incorrect answers. Sociodemographic variables included age, marital status, education, occupation, monthly income level, place of residence, gestational week, and parity; daily behaviors included diet, sleep, physical exercise, following news related to the coronavirus, and electronic screen exposure; family status included recent major incidents at home and family support; and coronavirus-related events included having acquaintances infected with coronavirus, incidence trend of coronavirus infections in the community they lived, and subjective life impact by the coronavirus.

The Generalized Anxiety Disorder-7 (GAD-7) scale was used to measure anxiety symptoms and their severity. The GAD-7 has been confirmed to be a reliable tool for assessing anxiety in the Chinese population and pregnant women (Tong et al., 2016; Sinesi et al., 2019). Seven items with typical anxiety symptoms over the past 2 weeks were measured on a 4-point Likert-type scale: 0 = never, 1 = several days, 2 = more than half of the days, and 3 = nearly every day. As in a recent study (Austin et al., 2021), the severity of anxiety was classified by the total score as follows: mild (5–9), moderate (10–14), and severe (15–21). A cutoff score of 5 or higher was considered to indicate anxiety symptoms. The Cronbach’s alpha of GAD-7 in this study was 0.89.

We used the Patient Health Questionnaire-9 (PHQ-9) to assess depression symptoms and their severity. The PHQ-9 has been previously used in the Chinese population and found to be reliable (Lu et al., 2018). Nine items about typical depression symptoms over the past 2 weeks were measured on a four-point Likert-type scale: 0 = never, 1 = several days, 2 = more than half of the days, and 3 = nearly every day. As in a previous study (Lu et al., 2018), the severity of depression was classified by the total score according to the following: mild (5–9), moderate (10–14), severe (15–19), and extremely severe (20–27). We defined a PHQ total score of five points or greater as the presence of depression symptoms (Lin et al., 2021). The Cronbach’s alpha of PHQ-9 in this study was 0.86.

### Sample Size

The sample size was determined via the formula *n* = αz/2*p*(1-p)δ2, using a αz/2 of 1.96, expected anxiety or depression prevalence (30%), and the admissible error (δ) of 4%. Based on this, the recommended sample size was 504 participants.

### Statistical Analysis

We used the mean and standard deviation (SD) to describe continuous variables and numbers and percentages to display categorical variables. The chi-square test was applied to analyze the prevalence of anxiety and depression symptoms across different groups. Multivariate logistic regression models were established to explore the potential risk factors for anxiety and depression symptoms. Odds ratios (OR) and 95% confidence intervals (95% CI) were also obtained. All data were analyzed by using SPSS 22.0 (IBM SPSS Statistics, New York, NY, United States). Two-tailed tests with a value of *p* less than 0.05 were considered statistically significant.

## Results

In total, 684 pregnant women in Changzhou were enrolled in our study, and all of them completely filled out the questionnaires. Three questionnaires were excluded because the time spent on them was less than 5 min. Of all participants, the mean score on the GAD-7 scale was 3.38 (SD: 3.54), and the mean score on the PHQ-9 scale was 4.17 (SD: 3.97). The prevalence rates of anxiety and depression symptoms were 31.72% (26.28% for mild, 3.96% for moderate, and 1.48% for severe symptoms) and 36.12% (25.55% for mild, 8.22% for moderate, 1.62% for severe, and 0.73% for extremely severe symptoms), respectively.

The prevalence rates of anxiety and depression symptoms as stratified by several potential influence factors are shown in Table 1. There was no statistically significant difference in the prevalence of anxiety symptoms by age, marital status, education level, occupation, monthly income, gestational week, parity, or recent adverse events (*p* > 0.05). However, participants with a lower education level, part of a migrant population, having an irregular diet, bad subjective sleep quality, perceptions of poor family support, no physical exercise, exposure to electronic screens for a long time, spending a long time on news related to the coronavirus, having acquaintances infected with coronavirus, and lives seriously impacted by the coronavirus had a significantly higher prevalence of anxiety symptoms (*p* < 0.05). Moreover, no statistically significant difference was found in the prevalence of depression symptoms by age, education level, occupation, monthly income, permanent population, gestational week, parity, recent adverse events, and having acquaintances infected with coronavirus (*p* > 0.05). Participants with single or divorced marital status, having an irregular diet, bad subjective sleep quality, perception of poor family support, no physical exercise, exposure to electronic screens for a long time, spending a long time on news related to the coronavirus, and lives seriously impacted by the coronavirus had a significantly higher prevalence of depression symptoms (*p* < 0.05).

**TABLE 1:** Distribution of factors between positive and negative groups and results of the chi-square test.

Variables	Anxiety symptoms				Depression symptoms			
	Negative	Positive	*χ^2^*	*p*	Negative	Positive	*χ^2^*	*p*
Age			1.43	0.23			0.03	0.86
<35	434 (68.89)	196 (31.11)			403 (63.97)	227 (36.03)		
≥35	31 (60.78)	20 (39.22)			32 (62.75)	19 (37.25)		
Marital status			2.71	0.10			**6.78**	**0.01**
Married	448 (68.92)	202 (31.08)			422 (64.92)	228 (35.08)		
Single/divorced	17 (54.84)	14 (45.16)			13 (41.94)	18 (58.06)		
Education			**8.73**	**0.03**			0.19	0.98
College or above	197 (73.78)	70 (26.22)			172 (64.42)	95 (35.58)		
Junior college	137 (66.83)	68 (33.17)			129 (62.93)	76 (37.07)		
High school	64 (67.37)	31 (32.63)			60 (63.16)	35 (36.84)		
Middle school or below	67 (58.77)	47 (41.23)			74 (64.91)	40 (35.09)		
Occupation			1.50	0.47			1.13	0.57
Healthcare worker	21 (70.00)	9 (30.00)			21 (70.00)	9 (30.00)		
Employed by other	331 (69.53)	145 (30.47)			307 (64.50)	169 (35.50)		
Unemployed	113 (64.57)	62 (35.43)			107 (61.14)	175 (38.86)		
Monthly income (RMB)			1.27	0.74			4.96	0.17
≥10,000	14 (63.64)	8 (36.36)			12 (54.55)	10 (45.45)		
≥6,000	61 (67.03)	30 (32.97)			56 (61.54)	35 (38.46)		
≥3,000	266 (70.00)	114 (30.00)			256 (67.37)	124 (32.63)		
<3,000	124 (65.96)	64 (34.04)			111 (59.04)	77 (40.96)		
Permanent population			**3.89**	**0.05**			2.99	0.08
Yes	447 (69.09)	200 (30.91)			418 (64.61)	229 (35.39)		
No	18 (52.94)	16 (47.06)			17 (50.00)	17 (50.00)		
Gestational week			0.84	0.66			0.47	0.79
<14	238 (69.59)	104 (30.41)			217 (63.45)	125 (36.55)		
≥14	63 (69.23)	28 (30.77)			56 (61.54)	35 (38.46)		
≥28	164 (66.13)	84 (33.87)			162 (65.32)	86 (34.68)		
Parity			1.99	0.37			2.31	0.31
1	307 (70.09)	131 (29.91)			273 (62.33)	165 (37.67)		
2	147 (65.33)	78 (34.67)			152 (67.56)	73 (32.44)		
3	11 (61.11)	7 (38.89)			10 (55.56)	8 (44.44)		
Adverse events			1.43	0.23*			1.19	0.27
No	458 (68.67)	209 (31.33)			428 (64.17)	239 (35.83)		
Yes	7 (50.00)	7 (50.00)			7 (50.00)	7 (50.00)		
Diet			**30.94**	**<0.01**			**40.38**	**<0.01**
Regular	427 (72.13)	165 (27.87)			405 (68.41)	187 (31.59)		
Irregular	38 (42.70)	51 (57.30)			30 (33.71)	59 (66.29)		
Subjective sleep quality			**90.26**	**<0.01**			**107.23**	**<0.01**
Good	298 (83.01)	61 (16.99)			289 (80.50)	70 (19.50)		
Ordinary	155 (55.76)	123 (44.24)			138 (49.64)	140 (50.36)		
Poor	12 (27.27)	32 (72.73)			8 (18.18)	36 (81.82)		
Support from family members			**34.31**	**<0.01**			**42.25**	**<0.01**
All-round	319 (76.50)	98 (23.50)			306 (73.38)	111 (26.62)		
Ordinary	114 (56.72)	87 (43.28)			97 (48.26)	104 (51.74)		
Very few	32 (50.79)	31 (49.21)			32 (50.79)	31 (49.21)		
Physical exercise per day (minutes)			**11.56**	**<0.01**			**25.92**	**<0.01**
<60	393 (71.20)	159 (28.80)			376 (68.12)	176 (31.88)		
≥60	9 (60.00)	6 (40.00)			10 (66.67)	5 (33.33)		
None	63 (55.26)	51 (44.74)			49 (42.98)	65 (57.02)		
Time of electronic screen exposure per day (hours)			**10.30**	**0.01**			**32.26**	**<0.01**
<3	148 (77.08)	44 (22.92)			150 (78.13)	42 (21.87)		
≥3	134 (67.00)	66 (33.00)			132 (66.00)	68 (34.00)		
≥5	183 (63.32)	106 (36.68)			153 (52.94)	136 (47.06)		
Time spent on news related to the coronavirus per day (hours)			**6.28**	**0.01**			**5.54**	**0.02**
<1	333 (71.31)	134 (28.69)			312 (66.81)	155 (33.19)		
≥1	132 (61.68)	82 (38.32)			123 (57.48)	91 (42.52)		
Having acquaintances infected with coronavirus			**5.91**	**0.02**			1.94	0.16
No	441 (69.45)	194 (30.55)			410 (64.57)	225 (35.43)		
Yes or uncertain	24 (52.17)	22 (47.83)			25 (54.35)	21 (45.65)		
Subjective life impact by the coronavirus			**37.30**	**<0.01**			**40.93**	**<0.01**
Mild	116 (82.86)	24 (17.14)			111 (79.29)	29 (20.71)		
Moderate	319 (68.00)	150 (32.00)			299 (63.75)	170 (36.25)		
Severe	30 (41.67)	42 (58.33)			25 (34.72)	47 (65.28)		

*Value of p was calculated by the method of successive correction.

*Bold values indicate statistically significant effect sizes.*

Influencing factors significantly associated with the anxiety and depression symptoms above were included in the multivariate logistic regression models. The above associations were weak but there was still a statistical difference. The associations of potential risk factors with anxiety and depression symptoms during the COVID-19 pandemic in pregnant women are presented in Tables 2, 3. In the multivariate logistic regression models, an irregular diet (OR = 2.57, 95% CI: 1.54–4.29), ordinary subjective sleep quality (OR = 3.23, 95% CI: 2.21–4.73), poor subjective sleep quality (OR = 7.02, 95% CI: 3.26–15.09), perceiving ordinary family support (OR = 1.89, 95% CI: 1.27–2.82), very little family support (OR = 2.34, 95% CI: 1.28–4.26), spending over 1 h per day on news related to the coronavirus (OR = 1.58, 95% CI: 1.07–2.32), and severe subjective life impacts by the coronavirus (OR = 3.50, 95% CI: 1.71–7.15) were significantly associated with anxiety symptoms. Similarly, an irregular diet (OR = 2.90, 95% CI: 1.68–5.01), ordinary subjective sleep quality (OR = 3.44, 95% CI: 2.36–5.02), poor subjective sleep quality (OR = 8.45, 95% CI: 3.50–20.43), perceiving ordinary family support (OR = 2.26, 95% CI: 1.52–3.38), no physical exercise (OR = 1.99, 95% CI: 1.21–3.28), exposure to electronic screens over 5 h per day (OR = 2.01, 95% CI: 1.26–3.22), spending more than 1 h per day on news related to the coronavirus (OR = 1.65, 95% CI: 1.11–2.44), and severe subjective life impacts by the coronavirus (OR = 3.60, 95% CI: 1.72–7.52) were significantly associated with depression symptoms in the multivariate logistic regression models.

**TABLE 2:** Multivariate logistic regression analysis of potential risk factors for anxiety symptoms.

Variable	β	_*S* ${}_{\bar{X}}$ _	Wald χ^2^	*p*	OR (95% CI)
**Diet**					
**Regular**					**Reference**
Irregular	0.95	0.26	13.08	**<0.01**	**2.57 (1.54, 4.29)**
**Subjective sleep quality**					
**Good**					**Reference**
Ordinary	1.17	0.19	36.69	**<0.01**	**3.23 (2.21, 4.73)**
Poor	1.95	0.39	24.85	**<0.01**	**7.02 (3.26, 15.09)**
**Support from family members**					
**All-round**					**Reference**
Ordinary	0.64	0.20	9.99	**<0.01**	**1.89 (1.27, 2.82)**
Very few	0.85	0.31	7.67	**0.01**	**2.34 (1.28, 4.26)**
**Time spent on news related to the coronavirus per day (hours)**					
<1					Reference
≥1	0.46	0.20	5.36	**0.02**	**1.58 (1.07, 2.32)**
**Subjective life impact by the coronavirus**					
Mild					Reference
Moderate	0.49	0.26	3.38	0.07	1.63 (0.97, 273)
Severe	1.25	0.36	11.82	**<0.01**	**3.50 (1.71, 7.15)**

*Bold values indicate statistically significant effect sizes.*

**TABLE 3:** Multivariate logistic regression analysis of potential risk factors for depression symptoms.

Variable	β	_*S* ${}_{\bar{X}}$ _	Wald χ^2^	*p*	OR (95% CI)
**Diet**					
**Regular**					**Reference**
Irregular	1.07	0.28	14.59	**<0.01**	**2.90 (1.68, 5.01)**
**Subjective sleep quality**					
**Good**					**Reference**
Ordinary	1.24	0.19	41.35	**<0.01**	**3.44 (2.36, 5.02)**
Poor	2.13	0.45	22.49	**<0.01**	**8.45 (3.50, 20.43)**
**Support from family members**					
**All-round**					**Reference**
Ordinary	0.82	0.20	15.98	**<0.01**	**2.26 (1.52, 3.38)**
Very few	0.43	0.32	1.83	0.18	1.54 (0.82, 2.89)
**Physical exercise per day (minutes)**					
<60					Reference
≥60	0.12	0.64	0.04	0.85	1.13 (0.32, 3.95)
None	0.69	0.25	7.36	**0.01**	**1.99 (1.21, 3.28)**
**Exposure to electronic screen per day (hours)**					
<3					Reference
≥3	0.39	0.26	2.32	0.13	1.48 (0.89, 2.44)
≥5	0.70	0.24	8.51	**<0.01**	**2.01 (1.26, 3.22)**
**Time spent on news related to the coronavirus per day (hours)**					
<1					
≥1	0.50	0.20	6.14	**0.01**	**1.65 (1.11, 2.44)**
**Subjective life impact by the coronavirus**					
Mild					Reference
Moderate	0.44	0.26	2.88	0.09	1.55 (0.93, 2.58)
Severe	1.28	0.38	11.62	**<0.01**	**3.60 (1.72, 7.52)**

*Bold values indicate statistically significant effect sizes.*

## Discussion

To our knowledge, there have been several studies investigating maternal mental health during the COVID-19 pandemic. Compared to studies conducted during the COVID-19 pandemic using the same mental measurements, the prevalence of anxiety symptoms in our study (31.72%) was close to those in Italy (32.6%) (Colli et al., 2021) and Ethiopia (32.2%) (Kassaw and Pandey, 2020), higher than those in Guangzhou (19.0%) (Zheng et al., 2021), Ireland (11%) (Farrell et al., 2020), and Warsaw (38%) (Nowacka et al., 2021). With regard to maternal depression, the prevalence of depression symptoms in our study was 36.12%, close to those in Shenzhen (35.6%) (Lin et al., 2021) and Guangzhou (35.3%) (Zheng et al., 2021), a little lower than that in central and western China (41.63%) (Zhou et al., 2021), and much lower than the rate (67.9%) in Iran (Vafaei et al., 2020). As a recent meta-analysis summarized, the prevalence of anxiety and depression symptoms among pregnant and postpartum women during the COVID-19 pandemic varies widely, fluctuating somewhere between 8 and 71% (Yan et al., 2020). The possible reasons for varied prevalence lay in diverse social, economic, cultural, and religious backgrounds in different countries and regions (Zhang et al., 2018). In addition, different cutoff scores for different measurement instruments (Lin et al., 2021) and distinct severity of the local pandemic across multiple studies could also contribute to these inconsistencies (Maffly-Kipp et al., 2021). Therefore, restrictive standard diagnosis tools, more reasonable cutoff scores, and multi-center investigations could be required in further studies. In addition, consideration should be given to routine mental health screening of emotionally sensitive groups during major health emergencies.

Discovering potential risk factors is helpful to guide the direction of preventive measures when encountering public health emergencies. To our knowledge, relatively more influencing factors have been included in our study to provide better suggestions. We found that anxiety and depression symptoms were more likely to occur in people with an irregular diet, bad subjective sleep quality, receiving less support from family members, spending more than 1 h per day on news related to the coronavirus, and lives impacted seriously by the coronavirus. Moreover, doing no physical exercise and exposure to electronic screens over 5 h per day were risk factors for depression symptoms. However, associations between marital status, education, occupation, monthly income, locality, gestational age, parity, having coronavirus infections among acquaintances, and anxiety or depression symptoms were not found in the multivariate logistic regression analyses.

A few studies have investigated diet and mental health during the COVID-19 pandemic, but previous studies found that adverse dietary habits, such as irregular eating times and reverse circadian rhythm, were risk factors for mental health (Godos et al., 2020). Therefore, healthy eating habits are necessary for preventing mental disorders. Similar to our study, Lin et al. (2021) showed that poor sleep conditions were strongly associated with gestational anxiety and depression symptoms. A possible reason is that the tired brain causes neurons to discharge abnormally, leading to certain neuropsychiatric symptoms (Aritake et al., 2015). Therefore, monitoring sleep quality and targeting interventions for improving sleep conditions might help alleviate adverse mental symptoms. As for family support, several recent studies have indicated that family support may help with reducing mental problems for pregnant women during the COVID-19 pandemic (Ceulemans et al., 2021; Nowacka et al., 2021; Zheng et al., 2021). A structural equation model that noted the lockdown policy has decreased maternal depression symptoms by enhancing family support (Zhou et al., 2021). Therefore, future policy should consider the impact of infection-reducing measures on the supporting role of family members during pregnancy. In our study, around a third of participants spent over 1 h per day on news related to the coronavirus. Another Chinese study showed that time spent focusing on the COVID-19 (≥3 h per day) was associated with anxiety symptoms (Huang and Zhao, 2020). Moreover, 42.44% of participants were exposed to electronic screens over 5 h per day, and this was a risk factor for depression symptoms. A study in India noted that increased total screen time was associated with higher depressive symptomatology (Majumdar et al., 2020). A Chinese study showed that increased maternal depression symptoms were found with increased internet use (Zhou et al., 2021). The association between the focus on the COVID-19 pandemic, electronic screen exposure, and mental disorders may have been attributed to misinformation, as has been very common in the pandemic era (Nowacka et al., 2021). In the context of COVID-19, the threat of the internet-induced panic warrants closer collaboration among media experts, health professionals, and policymakers to prevent an epidemic of misinformation (Killgore et al., 2020). In addition, we found respondents’ lives seriously impacted by the coronavirus were more likely to have psychological problems. Possible reasons are an inconvenience in obtaining antenatal examinations, lack of social and family support, and income losses because of a social isolation policy (Li et al., 2020; Cao et al., 2021). Consistent with our findings, many studies have demonstrated that physical exercise could reduce anxiety and depression symptoms in pregnant women and therefore could be an effective strategy to enhance mental health (Farrell et al., 2020; Colli et al., 2021).

There have been no agreed conclusions on the association of mental disorders with maternal age, marital status, education, occupation, income, gestational week, parity, and having coronavirus infections among acquaintances (Farrell et al., 2020; Ceulemans et al., 2021; Colli et al., 2021; Jelly et al., 2021; Shangguan et al., 2021). For example, contrary to our study, a study in Italy found increased levels of anxiety in unpartnered women (Colli et al., 2021). Single or divorced status is a condition that can increase the risk of anxiety, depression, and even suicide (Beutel et al., 2017). In addition, some researchers noted that working in healthcare may have protected women to some extent (Ceulemans et al., 2021), others found no association between occupation and mental impairment (Farrell et al., 2020), while Lai’s study indicated that medical health workers in fever clinics or hospital wards had more serious mental problems (Lai et al., 2020). In addition, Kassaw et al. found that primigravida status was a risk factor for perinatal anxiety (Kassaw and Pandey, 2020), while Yan et al. (2020) reported that multigravida women were more vulnerable to mental disorders. Moreover, a study in India revealed no association between anxiety and depression symptoms and gestational age (Farrell et al., 2020), while another Indian study reported the contrary conclusion (Jelly et al., 2021). The differences in these studies may be explained by the variation in the study population, different sample sizes, the distinct composition ratio of participants, and the different epidemic stages (the COVID-19 pandemic had been effectively controlled over 30 days after the outbreak, which probably eased the fear of the COVID-19 pandemic) (Zhou et al., 2020; Zhu and Zhu, 2021). Therefore, larger sample sizes, more related factors, such as skew covariates, analysis according to features of different subgroups, and meta-analysis of homogeneous participants need to be considered in future research (Irfan et al., 2021).

The unique feature of our manuscript is we included relatively more influencing factors, such as diet, having coronavirus infections among acquaintances, and so on. However, this study has some limitations. First, because of the cross-sectional design, the actual causality awaits further studies using a longitudinal design. Second, because of the voluntary participation option in online surveys, selection bias is inevitable, limiting the generalization of our findings to the whole population of pregnant women. Third, as GAD-7 and PHQ-9 are self-reported screening tools, the prevalence of anxiety and depression symptoms might be over- or underestimated to some extent, so diagnostic instruments are needed in future studies. Fourth, the study was produced in a relatively stable stage of the domestic pandemic, which may lead to an underestimation of the impact of the pandemic on maternal psychological symptoms and the potential risk factors. Fifth, according to different epidemic statuses of COVID-19 in distinct regions, our results could be used as a contemporary reference only for other cities with similar epidemic levels (51 cases among 5 million population) during the first phase of the COVID-19 pandemic.

## Conclusion

In conclusion, high prevalence rates of anxiety and depression symptoms are psychological consequences during outbreaks of the coronavirus. People with irregular diets, bad subjective sleep quality, receiving less support from family members, lives seriously impacted by the coronavirus, doing no physical exercise, and spending too much time exposed to electronic screens or reading news related to the coronavirus were at a high risk of displaying mental issues. Therefore, healthy eating habits, regular work and rest, proper exercise, disclosure of timely and accurate information, and providing family and social support are necessary measures to maintain maternal mental health. Mental healthcare from professional institutions should be directed to vulnerable people as early as possible when encountering public health emergencies.

## Data Availability Statement

The raw data supporting the conclusions of this article will be made available by the authors, without undue reservation.

## Ethics Statement

The studies involving human participants were reviewed and approved by the Ethics Committee of Changzhou Maternal and Child Health Care Hospital. The participants provided their written informed consent to participate in this study.

## Author Contributions

YZ and NY: analysis and interpretation of data, and drafting and revising of the manuscript. LW: study design and critical revision of the manuscript. HZ and XM: collection of data. All authors contributed to the final manuscript and submission.

## Conflict of Interest

The authors declare that the research was conducted in the absence of any commercial or financial relationships that could be construed as a potential conflict of interest.

## Publisher’s Note

All claims expressed in this article are solely those of the authors and do not necessarily represent those of their affiliated organizations, or those of the publisher, the editors and the reviewers. Any product that may be evaluated in this article, or claim that may be made by its manufacturer, is not guaranteed or endorsed by the publisher.
